# 

**Published:** 1915-11-27

**Authors:** 


### Institutional Pharmacists and Recruiting.

Pharmacists of military age who are engaged as dispensers at civil hospitals, infirmaries, and other institutions have, in common with other officials, received Lord Derby's recruiting letter, and no doubt this will be followed up by visits from canvassers. The Public Pharmacists' and Dispensers' Association has taken the matter up with the committee dealing with "reserved occupations," presided over by Lord Lansdowne. In requesting that pharmacists should be placed in the "starred" list, the Association points out that there is already a dearth of pharmacists (both men and women), and that if these officers are recruited it will be impossible to fill their places with suitably qualified persons. It is further suggested that an exemption badge should be granted, as with munition and other indispensable workers. The attitude now adopted by several hospital committees is that they have no power to refuse permission to any officer; all that can be done is to indicate to the authorities that certain of their officers are indispensable. These are advised to join up under the "Derby scheme," and their cases will be dealt with by the local tribunals. Whether this will prove satisfactory is doubtful, but it would appear to be the only way out for men who do not desire the stigma of "conscript" in the event of compulsory service being instituted. The Pharmaceutical Society has also made representations on the foregoing, and it is yet hoped to have pharmacists placed in the "starred" list.

### The Northern Association of Hospital Secretaries.

A meeting of the Association was held in the board-room of the Manchester Royal Infirmary on Saturday, November 20. Those present included Mr. W. G. Carnt, Mr. E. L. Blake, Mr. H. J. Dafforne, Mr. Ferguson, Mr. E. Forster, Mr. J. Nall, Mr. F. Oliver, Mr. A. Prophet, Mr. G. Ruddle, Mr. J. H. Shaw, Mr. N. A. Smith, and Mr. W. Stevenson. The chief item on the agenda was the resignation of the president, Mr. W. G. Carnt, which was accepted by the members with tokens of sincere sorrow. Mr. Carnt has held the presidency of the Association since its inception eight years ago, and he has been the means of bringing together the representatives of hospitals from Derby to Leeds for mutual benefit and brotherly co-operation and support. His resignation is a great blow not only to himself, but to all those who have for so many years received of his practical sympathy and wise counsels. Mr. G. Ruddle, secretary of Salford Royal Hospital, was unanimously elected president in Mr. Carnt's stead. The Association considers itself fortunate in having found so able a successor to their first president, and they have every reason to believe that the work so nobly and well done by Mr. Carnt will be continued by Mr. Ruddle. Mr. H. J. Dafforne, secretary of Ancoats Hospital, Manchester, was elected honorary secretary to the association in the place of Mr. H. J. Eason, who was appointed secretary to the London Lock Hospital. The members afterwards inspected the radium department, and spent a most enjoyable and instructive half-hour. Mr. Carnt kindly entertained the members to tea.

### PASSING EVENTS & TOPICS.

### The "Ninian Patrick Bed" at Cardiff.

Lord Ninian Crichton-Stuart, late M.P. for Cardiff, recently killed on active service, has left £1,000 to the King Edward VII.'s Hospital to endow a bed in the children's ward, to be called the "Ninian Patrick Bed," in memory of Lord and Lady Ninian's son, this endowment to take the place of the annual gift which Lord Ninian had subscribed during his life for the maintenance of such a bed.

### A Uniform System of Records.

At the hospitals for the observation and treatment of officers suffering from the stress and horrors of war rather than from wounds, it is gratifying to learn that the full clinical records are being kept upon a common plan, on certain printed forms supplied by the Medical Research Committee of the National Health Insurance. These records will, therefore, have a great scientific value apart from their historical or military significance.

### Public Library as War Hospital.

The Public Libraries Committee of the Islington Borough Council has recommended that the Council should permit the North Library, Manor Gardens, to be used as a hospital for wounded soldiers, subject to the condition that the Great Northern Central Hospital will undertake to pay all expenses connected with the adaptation, maintenance, and reinstatement of the building.

### The Laudanum Habit.

The action of the General Medical Council in recommending to the Privy Council that preparations of opium containing 0.75 per cent. of morphine should be included in Part 1 of the Poisons Schedule is in accord with public policy. The laudanum of the new Pharmacopœia contains a proportion of morphine which automatically brings it into the first part of the Schedule, and purchasers of this preparation are bound to sign the poison book. But undoubtedly a considerable business continues to be done in the weaker laudanum of the old Pharmacopœia, which can be sold by chemists with comparatively little restriction. It is not in the best interests of the public that this state of affairs should be allowed to go on, and no doubt the Pharmaceutical Society will receive a communication on the subject from the Privy Council in due course.

### Crutches for the Wounded.

With reference to the paragraph in our issue of November 13 on "Hospital Wood-Carving" a correspondent, who is in charge of a workroom where articles of woodwork and carpentry are being made and supplied to the local hospitals, writes: "I have been wondering this last few days whether you could ascertain if there is any demand for crutches for soldiers, either for temporary or permanent use, and whether we should be doing anyone a real service by making such things at the cost price of the wood and carriage to destination? I don't wish to undertake the work unless either the soldier himself or the Government would benefit by our doing it, but if there is any demand under those conditions I think I can get some ash wood at a cheap rate suitable for the purpose. If other work keeps coming in I could only supply one or two per week unless I can increase the number of helpers." If any of our readers know of cases where the supply of such crutches would fulfil the object aimed at, we shall be glad to forward their letters to our correspondent. All communications should be addressed to the Editor, The Hospital, 28 and 29 Southampton Street, Strand, and marked "Crutches" in the left-hand top corner.

### Gifts and Bequests.

The Worshipful Company of Salters have again sent a contribution of ten guineas to the Mental After-Care Association, Church House, Dean's Yard, Westminster.

Ancoats Hospital, Manchester, has received from Mr. R. F. Riddick and family, of Ashley, near Altrincham, Cheshire, the sum of £500 to endow a cot in the children's wards in memory of their daughter. The inscription to be placed on the cot is, "Agnes Riddick, September 26, 1915." Lieut. Chas. S. Haslam, of Dunham Knoll, Altrincham, has also sent to the treasurer of Ancoats Hospital a sum of £500 to endow a cot in the children's wards in memory of this lady. In addition the hospital has received a donation to the amount of £100 from the relatives of the late Mr. Wm. Norquoy, who died intestate, they being fully assured that he intended to leave a legacy to Ancoats Hospital. The donation is accompanied with the request that it be used for a definite object with which the late Mr. Norquoy's name can be associated by means of a brass tablet. Ancoats Hospital has also received, through the kindness of Mr. A. B. Harvey, of Lloyds Bank, King Street, Manchester, a trustee under the will of the late Mrs. Gregson, £500 worth of War Loan to endow a cot, the plate to be inscribed "The Elizabeth Gregson Cot, 1915," whilst the employees in the light gun shop department of Messrs. Armstrong, Whitworth and Co., of Openshaw, have given £50 for the purchase of an up-to-date vertical *x*-ray screen stand.

### A Curious Story.

Once upon a time the secretary of a hospital drafted and published an original advertisement appealing for funds. The advertisement in question did not meet with the approval of a certain highly-placed personage in the hospital world, and he wrote a very bitter letter to the secretary, a letter that was quite startling in its veiled threats and in the terrific force of its arguments. No doubt the highly-placed personage imagined that his letter would call forth the humble regrets and apologies of the hospital secretary, but such was not the case. All that the highly-placed personage received from the humble and lowly secretary was the following note: "Your letter of the inst. is very bloodthirsty. I suggest that a dagger would be of more use to you than a pen. When you care to submit suggested appeals to me for approval I shall be happy to return the compliment."

### Obituary.

Mr. F. Daly, L.R.C.S. Edin., Chairman of the Tottenham Bench of Magistrates for nearly thirty years, has died at Stoke Newington.

DEATH OF A MEDICAL OFFICER OF HEALTH.

The death is announced of Mr. Charles William Thorp, F.R.C.S. (Ireland). For thirty-seven years Mr. Thorp had been medical officer of health for Todmorden.

DEATH OF A MEDICAL EX-MAYOR.

The death has occurred of Dr. D. W. Brown, a former Mayor of Preston. Before entering on his medical studies at Edinburgh he had been a bank clerk at Manchester. Every phase of public health work commanded his attention, and especially the problem of the high infant mortality for which some years ago Preston had an unenviable notoriety.

Rates of Subscription: Twelve months, 6/6; Six months, 4/-; Three months, 2/-, post free to any address in the United Kingdom. Abroad, Twelve months, 8/8; Six months, 5/-; Three months, 2/6, post free.—Address The Subscription Dept., "The Hospital," The Hospital Building, 28/29 Southampton Street, Strand, London, W.C.

